# Patient Engagement Strategies for Women with Gestational Diabetes Mellitus in Low-Resource Community Health Settings: A Narrative Review and Clinical Principles

**DOI:** 10.3390/healthcare14172860

**Published:** 2026-09-05

**Authors:** Matthew P. Martin, Nina Russin, Misha Pangasa

**Affiliations:** 1 National Safety Net Advancement Center, College of Health Solutions, Arizona State University, Phoenix, AZ 85004, USA; 2 Department of Obstetrics and Gynecology, Valleywise Health, Phoenix, AZ 85008, USA

**Keywords:** gestational diabetes mellitus, patient engagement, patient-centered care, stepped care pathway, digital health, self-management

## Abstract

**Highlights:**

**What are the main findings?**

- Evidence supports patient engagement strategies that combine whole-person assessment, structured education, self-management support, digital health, and coordinated postpartum follow-up across the gestational diabetes care continuum.
- A practical stepped clinical care pathway is proposed to help low-resource community health settings implement evidence-supported, patient-centered care for women with gestational diabetes mellitus.

**What are the implications of the main findings?**

- Community health clinics may strengthen GDM care by adapting evidence-informed strategies such as culturally responsive education, digital support, active clinician feedback, and coordinated follow-up to local capacity.
- Future research should prioritize implementation studies, culturally tailored interventions, and strategies to improve postpartum engagement and long-term diabetes prevention in underserved populations.

**Abstract:**

**Background/Objectives**: Gestational diabetes mellitus (GDM) is one of the most common pregnancy complications and requires sustained patient engagement to achieve optimal maternal and neonatal outcomes. This narrative review synthesized current evidence on patient engagement strategies and developed clinical principles for implementing patient-centered care across the GDM care continuum. **Methods**: A domain-based narrative review was conducted using the Consensus literature search platform. A total of 5359 records were identified. After multi-phase screening for relevance and eligibility, 37 studies, including systematic reviews, randomized controlled trials, observational studies, and qualitative research, were included. Evidence was synthesized across four domains: universal screening and linkage to care, whole-person assessment, digital patient engagement, and postpartum transition. **Results**: Universal screening consistently detected more women with GDM than risk-based approaches, although greater detection alone did not consistently improve maternal or neonatal outcomes. Evidence supported structured patient education, culturally responsive communication, self-management support, and active clinician feedback as key components of effective engagement. Digital health interventions, including telehealth, mobile applications, and remote monitoring, improved adherence, self-management, patient satisfaction, and, in many studies, glycemic control when integrated with clinician oversight. Postpartum follow-up remained a persistent gap despite evidence supporting reminder systems and coordinated care transitions. These findings informed a stepped clinical care pathway tailored to low-resource community health settings. **Conclusions**: Current evidence supports several components of patient-centered GDM care that informed a proposed stepped clinical care pathway integrating early identification, whole-person assessment, structured education, self-management support, digital engagement, and coordinated postpartum care. Many recommended strategies can be implemented using existing personnel and low-cost digital technologies, although additional implementation research is needed to evaluate culturally tailored interventions and long-term effectiveness in underserved populations.

## 1. Introduction

### 1.1. Gestational Diabetes Mellitus Is a Growing Public Health Challenge

Gestational diabetes mellitus (GDM), defined as glucose intolerance first recognized during pregnancy, is one of the most common medical complications of pregnancy worldwide [1,2]. The International Diabetes Federation estimates that approximately one in five pregnancies is affected by hyperglycemia, with nearly 80% of these cases attributable to GDM, corresponding to a global prevalence of approximately 10–25% depending on the population and diagnostic criteria used [3].

In the United States, GDM affects approximately 5–10% of pregnancies using traditional diagnostic criteria, with higher estimates reported under the International Association of Diabetes and Pregnancy Study Groups (IADPSG) criteria [1]. The prevalence of GDM continues to increase worldwide, driven by rising rates of obesity, delayed childbearing, and other metabolic risk factors, making GDM an important public health concern with implications for both maternal and long-term cardiometabolic health [1,3,4].

The burden of GDM is disproportionately borne by racial and ethnic minority populations and women experiencing socioeconomic disadvantage. Hispanic, Asian, American Indian/Alaska Native, and some Black populations experience higher rates of GDM, particularly in the presence of obesity, advanced maternal age, and adverse social determinants of health [3,5,6].

Geographic disparities, food insecurity, limited healthcare access, language barriers, and reduced access to culturally appropriate education further contribute to inequities in diagnosis, treatment, and outcomes [6]. These disparities underscore the need for patient-centered care models that address both clinical management and the social, cultural, and structural factors influencing patient engagement throughout pregnancy [7].

### 1.2. Consequences of Gestational Diabetes Mellitus

GDM has important short- and long-term consequences for both mother and child. During pregnancy, women with GDM typically experience increased insulin resistance and have increased risks of hypertensive disorders of pregnancy, including preeclampsia, cesarean delivery, preterm birth, and other obstetric complications [1,4,8]. Beyond pregnancy, GDM serves as an early marker of future cardiometabolic disease, substantially increasing the lifetime risk of recurrent GDM, type 2 diabetes mellitus, and cardiovascular disease [4,9]. Neonates born to mothers with GDM face increased risks of fetal overgrowth (macrosomia), shoulder dystocia and birth trauma, neonatal hypoglycemia, respiratory complications, hyperbilirubinemia, and admission to neonatal intensive care units [1,4,8].

Importantly, these consequences frequently extend beyond the perinatal period. Children exposed to intrauterine hyperglycemia have elevated risks of obesity, dyslipidemia, hypertension, impaired glucose metabolism, and type 2 diabetes later in life, contributing to an intergenerational cycle of metabolic disease [3,10]. Because many of these complications are preventable through timely diagnosis, effective glycemic control, and sustained postpartum follow-up, optimizing GDM management represents a critical opportunity to improve maternal and child health across the life course [1,4].

### 1.3. Why Patient Engagement Matters in GDM

Unlike many pregnancy complications that are managed primarily through clinician-directed interventions, successful GDM management depends heavily on the patient’s ability to perform daily self-management activities. Women diagnosed with GDM are expected to monitor blood glucose, modify their diet, increase physical activity, adhere to medication when indicated, attend frequent prenatal visits, and complete postpartum diabetes screening [1,2].

Successful self-management is influenced by multiple patient, provider, and health system factors. Healthcare providers consistently identify health literacy, self-efficacy, motivation, medication access, competing life demands, and fragmented care as important determinants of engagement, particularly among underserved populations [11].

Likewise, women with GDM report evolving informational, emotional, and practical needs throughout pregnancy and the postpartum period, underscoring the importance of structured education, family support, coordinated care, and patient-centered communication [7,12]. Consequently, improving maternal and neonatal outcomes requires patient engagement strategies that extend beyond clinical treatment to support sustained self-management across the continuum of care [7,11,13].

### 1.4. Unique Challenges Across Resource-Constrained Health Settings

Women receiving GDM care in resource-constrained settings often face multiple barriers that limit their ability to engage in recommended self-management. Transportation difficulties, competing work schedules, childcare responsibilities, food insecurity, financial constraints, language barriers, and limited health literacy can reduce adherence to glucose monitoring, dietary recommendations, medications, clinic visits, and postpartum follow-up [6,7,11]. Women consistently report the need for culturally appropriate education, individualized dietary guidance, clear communication, and greater emotional and family support throughout pregnancy and the postpartum period [7,12].

These patient-level barriers are often compounded by health system constraints, including workforce shortages, limited access to diabetes educators and specialists, and fragmented coordination between obstetric, primary care, and postpartum services [7,11]. Importantly, resource constraints occur across different healthcare contexts and should not be treated as uniform. In high-income countries, resource-constrained community health centers, safety-net systems, and rural clinics may operate within comparatively well-developed healthcare infrastructure while still experiencing workforce shortages, limited specialty access, financial constraints, and fragmented referral systems. Low- and middle-income country settings may face additional or more pronounced limitations related to healthcare financing, workforce availability, laboratory and medication access, digital infrastructure, and referral capacity. These contextual differences influence which patient engagement strategies are feasible, scalable, and sustainable.

Across settings, resource constraints can contribute to gaps in postpartum glucose testing and long-term follow-up despite the elevated risk of future type 2 diabetes [14]. Because women in underserved communities frequently experience multiple barriers simultaneously, improving patient engagement requires strategies that address both clinical care and social determinants of health while accounting for local workforce, infrastructure, financing, and care-delivery capacity [6,11,12]. Accordingly, the clinical principles proposed in this review should be viewed as adaptable strategies rather than a uniform implementation model across all resource-constrained settings.

### 1.5. The Evidence Gap

Over the past decade, a growing body of research has examined interventions intended to improve GDM management, including screening strategies, culturally tailored education, telehealth, mobile health applications, remote glucose monitoring, multidisciplinary care, and postpartum follow-up [15,16,17]. However, this evidence remains dispersed across multiple disciplines and intervention types, with most reviews focusing on individual components of care rather than patient engagement across the full continuum of GDM management.

As a result, clinicians and healthcare systems lack a practical synthesis of evidence that integrates screening, whole-person assessment, self-management support, digital engagement, and postpartum care into a patient-centered framework. Furthermore, relatively little attention has been given to how these strategies can be implemented within low-resource community health settings, where barriers to engagement are often greatest.

### 1.6. Purpose of This Review

The purpose of this narrative review is to synthesize current evidence regarding patient engagement strategies across the continuum of GDM care—from diagnosis through postpartum follow-up—with an emphasis on approaches that are feasible for implementation in low-resource community health settings. Specifically, this review examines evidence related to whole-person assessment, patient education, self-management support, digital engagement, and postpartum transition and proposes practical clinical principles to improve patient engagement and maternal and neonatal outcomes.

## 2. Materials and Methods

### 2.1. Review Design

This structured, domain-based narrative review synthesized current evidence supporting patient engagement strategies across the continuum of gestational diabetes mellitus (GDM) care, with particular emphasis on approaches applicable to low-resource community health settings. The review sought to synthesize evidence across the GDM care continuum and use the resulting findings to inform development of a proposed stepped clinical care pathway integrating early identification, whole-person assessment, patient education, self-management support, digital engagement, and postpartum follow-up.

A structured narrative review design was selected because the objective was to integrate heterogeneous evidence across multiple components of the GDM care continuum rather than evaluate the effectiveness of a single intervention or estimate a pooled treatment effect. The review incorporated evidence from diverse study designs addressing clinical effectiveness, patient experiences, behavioral and psychosocial factors, digital engagement, care delivery, and implementation. This approach allowed clinical and implementation evidence to be synthesized within a common patient-engagement framework while retaining the broader interpretive scope appropriate to a narrative review.

### 2.2. Information Sources and Search Strategy

The literature search was conducted on 3 June 2026, using the Consensus literature search platform [18], which indexes scientific publications from PubMed, Semantic Scholar, Crossref, and other scholarly sources. No lower publication-date restriction was applied; searches included the English-language, peer-reviewed literature published through the date of the search. Consensus was selected as the primary literature identification platform because it supports semantic searching, query expansion, citation-based discovery, and evidence synthesis across multiple study designs. The platform was used to facilitate literature identification; however, all study selection, eligibility decisions, data extraction, study verification, and narrative synthesis were performed by the first author and reviewed by the second author.

An author-developed Deep Search prompt specified the population of interest, four evidence domains, study designs of interest, areas of uncertainty, and an emphasis on implementation in low-resource settings. The four domains were: (1) screening and early identification; (2) whole-person assessment of social, cultural, language, psychosocial, and access barriers; (3) patient engagement strategies, including digital health, telehealth, remote monitoring, and self-management interventions; and (4) postpartum transition and type 2 diabetes prevention. Consensus subsequently decomposed the initial prompt into targeted natural-language search queries designed to increase the specificity and breadth of literature retrieval.

Search expansion included alternative terminology, clinical guidelines and foundational care models, citation-based discovery, and targeted searches addressing implementation in low-resource settings. To capture conflicting as well as supportive evidence, additional searches explicitly targeted limitations, barriers, null findings, and mixed evidence related to GDM screening, digital interventions, and postpartum care. Citation-graph expansion was also used to identify additional potentially relevant publications. The original Deep Search prompt and natural-language search queries generated during the search process are available in Supplementary Table S1.

### 2.3. Study Selection and Eligibility Criteria

The domain-based literature searches identified 5359 potentially relevant records. Records underwent title and abstract screening for relevance to the review objectives and predefined eligibility criteria. Duplicate records identified across searches were automatically removed by Consensus during the study-selection process before full-text assessment. Studies were retained for full-text assessment when they addressed GDM and provided evidence relevant to patient engagement, care delivery, or implementation across the GDM care continuum.

Following title and abstract screening, 50 reports were retained for full-text assessment. Full texts were evaluated for alignment with the review objectives and eligibility criteria. Preference was given to systematic and scoping reviews, randomized controlled trials, observational studies, and qualitative or mixed-methods research. Narrative reviews and intervention-development studies were included selectively when they provided relevant clinical, behavioral, or implementation evidence not adequately represented by other study designs. Studies focused primarily on disease pathophysiology, molecular mechanisms, genetics, or basic epidemiology without direct relevance to patient engagement or care delivery were excluded.

**Figure 1:**
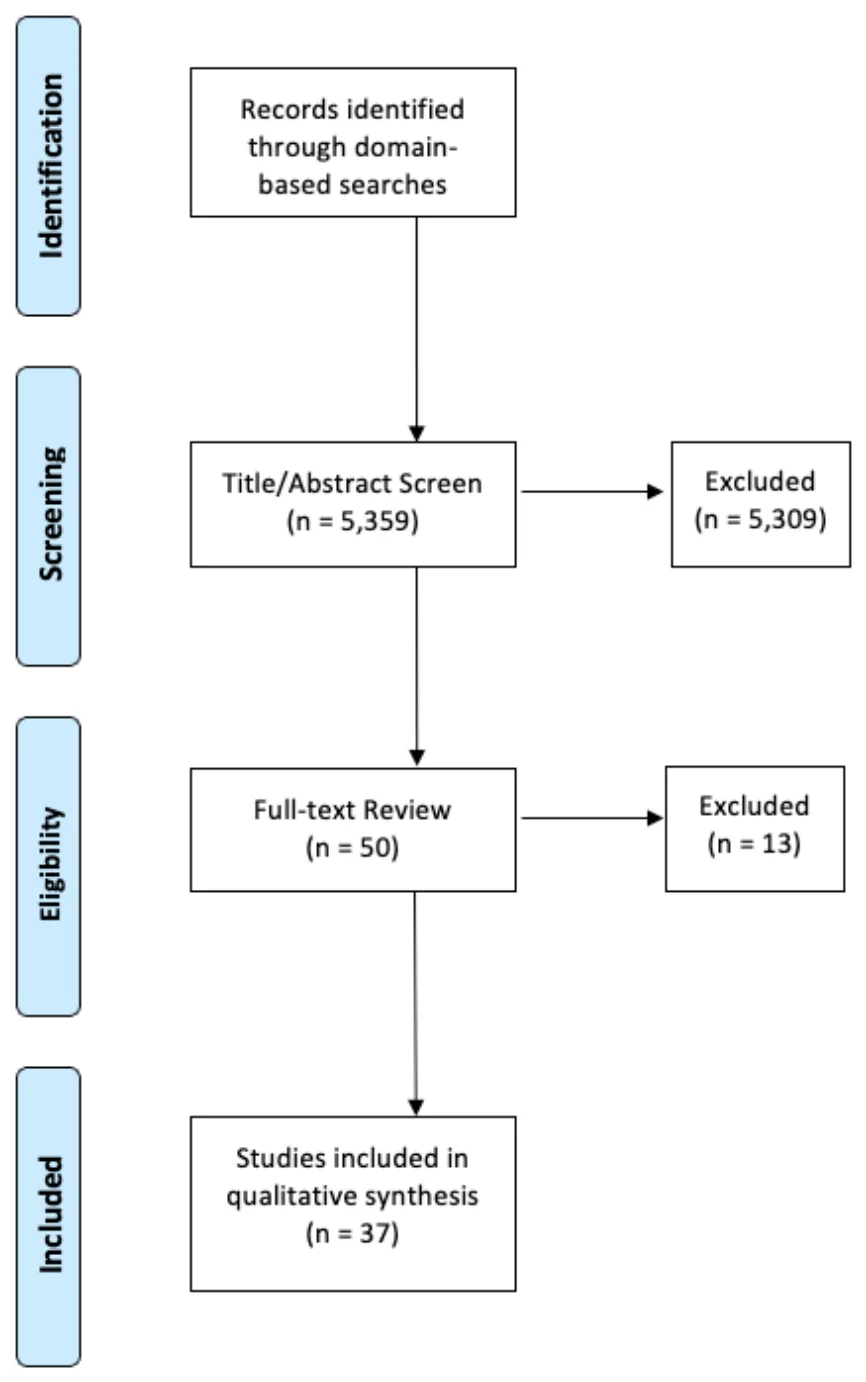
Literature Identification and Study Selection Process.

Following full-text assessment, 13 reports were excluded, resulting in 37 studies included in the structured narrative synthesis. A PRISMA-style flow diagram was used to transparently summarize literature identification, screening, eligibility assessment, and final study selection (Figure 1).

### 2.4. Data Extraction and Human Verification

All studies identified for inclusion underwent independent full-text review by the first author to verify relevance, study design, intervention characteristics, and key findings. Information extracted from each study included study setting, design, population, intervention or focus, and principal findings relevant to patient engagement across the stepped clinical care pathway. To improve consistency and reduce extraction errors, the second author independently reviewed a randomly selected subset of included studies and compared extracted data with the first author’s results. Discrepancies were resolved through discussion and consensus before finalizing the evidence tables.

### 2.5. Evidence Synthesis

For each included study, the authors extracted study characteristics (country, study design, population, intervention or focus, and principal findings) into a standardized evidence table that informed the narrative synthesis and development of clinical principles. Evidence was synthesized according to the four pathway domains rather than by study design. Because the objective was to identify evidence supporting implementation of patient engagement strategies rather than evaluate a single intervention, qualitative, mixed-methods, and implementation studies were considered complementary to randomized trials in informing the proposed stepped clinical care pathway. Findings were subsequently translated into evidence-informed clinical principles intended to support implementation within low-resource community health settings.

Because this structured narrative review incorporated heterogeneous study designs across multiple clinical and implementation domains, a formal study-level risk-of-bias assessment using standardized instruments was not conducted. Instead, the authors characterized the nature of the evidence supporting each proposed clinical principle according to the types and consistency of supporting studies. These descriptive classifications were author-developed and were not intended to represent formal methodological-quality assessments or certainty-of-evidence ratings such as GRADE. Accordingly, conclusions were interpreted in relation to study design, consistency of findings, and the degree to which evidence directly addressed the proposed patient-engagement strategy.

## 3. Results

### 3.1. Overview of Findings

The literature search identified 5359 potentially relevant publications. After screening for relevance, eligibility, and alignment with the review objectives, 37 studies were included in the final synthesis. The evidence base included systematic reviews and meta-analyses, randomized controlled trials, cohort studies, implementation studies, qualitative and mixed-methods research, and narrative reviews.

The included studies addressed four domains of a stepped clinical care pathway: (1) early identification of GDM through screening, (2) whole-person assessment of psychosocial, cultural, and social determinants of health, (3) patient engagement through digital health and self-management support, and (4) postpartum transition and type 2 diabetes prevention.

Table 1 summarizes the characteristics of the 37 included studies published between 2000 and 2026. The evidence base spanned all four domains, with the greatest concentration of studies addressing screening and early identification and patient engagement strategies. Systematic and scoping reviews were the most common study designs, followed by randomized controlled trials and cohort or observational studies. More than half of the included studies were international or multinational, reflecting evidence synthesized or collected across multiple countries. Among studies conducted in individual countries, the United States contributed the largest number, with the remaining studies representing diverse geographic settings.

Table 2 provides a detailed overview of the included studies, including study design, evidence domain, intervention or area of focus, and principal findings. The studies span the continuum of gestational diabetes care, from screening and diagnosis to digital health interventions, postpartum transition, and patient engagement strategies.

### 3.2. Step 1. Screening, Early Identification, and Prompt Linkage to Care

Evidence regarding GDM screening addresses three related but distinct questions: whether screening should be universal or risk-based, whether a one-step or two-step diagnostic approach should be used, and whether screening earlier than the standard 24–28-week period improves outcomes. Universal screening consistently identifies more women with GDM than risk-based approaches, while selective screening may miss a substantial proportion of cases [27,33,42,49]. However, greater case detection does not by itself establish improved maternal or neonatal outcomes, and the clinical implications depend on subsequent treatment and follow-up.

Studies comparing one-step and two-step approaches similarly demonstrate differences in GDM detection, largely reflecting differences in diagnostic criteria and thresholds [25,29,30,31,37,44]. One-step approaches generally identify more women with GDM, but randomized and observational studies have not demonstrated consistent improvements in maternal or neonatal outcomes [25,29,30,31,37,44]. Some studies report potential benefits for selected outcomes, whereas others identify increased diagnoses without corresponding reductions in maternal or neonatal morbidity. Early-pregnancy screening represents a separate question. Although earlier screening may identify women with hyperglycemia or elevated GDM risk sooner, current evidence has not demonstrated consistent improvements in maternal or neonatal outcomes compared with screening at the guideline-recommended period [19,46]. Studies conducted during the COVID-19 period also demonstrate how screening and diagnostic strategies may be modified in response to healthcare constraints; alternative criteria increased GDM identification in some settings without substantial changes in pregnancy outcomes [34,45]. Beyond conventional screening strategies, emerging precision approaches using routinely available clinical measures may help identify women more likely to require pharmacologic treatment, although their role in routine care remains to be established [22].

### 3.3. Step 2. Whole-Person Assessment

Across qualitative, mixed-methods, and implementation studies, barriers to effective engagement consistently include limited knowledge about GDM before pregnancy, time constraints, caregiving responsibilities, financial hardship, language and cultural discordance with healthcare providers, inadequate social support, fragmented communication, and competing work and family demands [20,40,41,43,50].

Conversely, strong family and community support, higher self-efficacy, culturally tailored education, patient-centered communication, and coordinated multidisciplinary care consistently facilitate greater adherence to recommended self-management behaviors [20,40,50]. Health system barriers—including fragmented referral pathways, inconsistent provider communication, and poor integration between obstetric and primary care services—further impede effective management, particularly during the transition to postpartum care [32].

Across these studies, women’s informational, psychosocial, cultural, and practical needs varied across the GDM care continuum, supporting individualized rather than uniform approaches to education and self-management support [20,35,40,43]. Culturally appropriate communication, family involvement, and continuity of care were recurrent themes associated with greater engagement [20,40,50]. Intervention-development research further illustrates how these findings can inform care design. The IINDIAGO intervention used formative evidence and a behavior-change framework to incorporate peer counseling, diabetes nursing, psychosocial support, and postpartum follow-up in response to identified patient needs [39].

### 3.4. Step 3. Patient Engagement Through Digital Support

Patient engagement strategies extend beyond technology and include collaborative care, patient education, and support for adherence to recommended management [38]. Digital health represents one approach to reinforcing these activities. Digital health interventions for GDM include several distinct approaches with different functions and outcomes. SMS reminders and educational messaging primarily support adherence, self-monitoring, and patient education, although benefits may be smaller when baseline adherence is already high [16]. Mobile applications and web-based educational tools can support knowledge, goal setting, self-management, and engagement and, in some studies, are associated with improved glycemic control [23,28,36,48]. However, usability, digital health literacy, technical barriers, privacy concerns, and limited cultural tailoring may reduce their accessibility and effectiveness, particularly for underserved populations [23,48]. Digital tools may also support individualized self-management approaches by helping patients relate glucose patterns to behavioral strategies such as nutrition and meal timing, although evidence for specific approaches such as chrononutrition remains emerging [51].

Telehealth encounters can improve access, convenience, patient–provider communication, and continuity of care while reducing the need for some in-person visits [21,28,52,53]. Remote glucose monitoring, in contrast, enables transmission of patient-generated glucose data for clinician review, feedback, and treatment adjustment. Interactive approaches that connect monitoring with timely clinical feedback may facilitate earlier treatment escalation and improve self-management, whereas passive data collection alone offers less clear benefit [52].

Overall, evidence for digital interventions is the strongest for engagement and process outcomes, including adherence, glucose-monitoring behaviors, convenience, communication, and patient satisfaction. Improvements in glycemic control have also been reported, but effects vary across intervention types and study populations. Evidence for consistent improvements in maternal and neonatal outcomes is less definitive [13,16,21,23,28,36,48,52]. Accordingly, digital interventions should be selected according to their intended function and should complement, rather than replace, patient–provider interaction.

### 3.5. Step 4. Coordinated Postpartum Transition and Type 2 Diabetes Prevention

Sustained patient engagement beyond delivery is essential because women with prior GDM remain at substantially increased lifetime risk of developing type 2 diabetes. Nevertheless, postpartum follow-up remains one of the weakest components of contemporary GDM care. Across systematic reviews and primary studies, postpartum glucose testing remains suboptimal, with many women not completing recommended follow-up [14,24,32]. Longer-term follow-up is similarly underdeveloped. A scoping review identified no established systematic cardiovascular follow-up strategy after GDM and found no consistent advantage of eHealth approaches over conventional follow-up [26].

Barriers to successful postpartum follow-up include limited awareness of future diabetes risk, competing childcare and work responsibilities, transportation challenges, fragmented transitions from obstetric to primary care services, and inadequate provider communication and follow-up systems [14,32,47]. Qualitative evidence also indicates dissatisfaction with postnatal care when information and advice are inconsistent [47].

Several interventions have demonstrated modest improvements in postpartum engagement. Integrating maternal diabetes follow-up with pediatric well-child visits, implementing proactive reminder systems using telephone calls or SMS messaging, and establishing mother–infant dyad care models increase postpartum attendance and diabetes screening rates, although these approaches require organizational resources that may be unavailable in lower-resource settings [14,24].

Long-term prevention efforts after GDM are challenged by low uptake and adherence, highlighting the importance of continued education, social support, and active follow-up [40]. Collectively, these findings suggest that postpartum planning should begin during pregnancy and continue through coordinated primary care follow-up, representing the final—and frequently weakest—step in the GDM care pathway. Table 3 summarizes evidence-supported patient engagement strategies identified in this review and their application across each step of a GDM care pathway.

### 3.6. Summary of Findings

Across the 37 included studies, evidence was identified for each component of a stepped clinical care pathway for GDM, including screening and diagnosis, whole-person assessment, structured education, self-management support, digital engagement, and postpartum transition. The evidence was most consistent for increased case detection with universal screening and for improvements in selected engagement and self-management outcomes with digital and behavioral strategies, whereas evidence directly evaluating whole-person and postpartum engagement models was more limited. Few studies evaluated the proposed integrated pathway as a whole.

## 4. Discussion

### 4.1. Principal Findings

This review demonstrates that although multiple evidence-supported patient engagement strategies exist across the continuum of GDM care, few studies have evaluated these interventions as components of an integrated stepped clinical care pathway. Instead, most investigations focused on individual strategies rather than coordinated models of care. The screening evidence illustrates an important distinction between case detection and clinical benefit. Screening approaches influence whom and how many women are diagnosed with GDM, but increased detection has not consistently translated into improved maternal or neonatal outcomes. From a patient-engagement perspective, screening therefore represents an entry point to care rather than an intervention in isolation; its clinical value depends on whether diagnosis is followed by timely communication, education, self-management support, appropriate treatment, and follow-up.

Across the remainder of the care continuum, effective patient engagement requires structured education, self-management support, active clinician feedback, and coordinated postpartum follow-up. However, implementation evidence remains limited, particularly for resource-constrained community health settings where patient engagement barriers may be greatest.

### 4.2. Implications for Resource-Constrained Community Health Settings

For resource-constrained community health settings, improving GDM care requires attention not only to patient engagement strategies but also to the organizational capacity needed to implement them. Standardized, multidisciplinary pathways may improve care coordination, self-management support, and efficiency, although effects on maternal and neonatal outcomes are more variable [50,54,55]. Successful implementation also depends on clearly defined team roles, training and supervision, communication, and organizational leadership [56,57].

Community health workers (CHWs), patient navigators, nurses, and other team members may support patient education, appointment attendance, care coordination, and navigation of social and healthcare barriers [58,59,60]. However, these strategies are not resource-neutral and require adequate staffing, role definition, clinical supervision, and sustainable workflows. Similarly, multidisciplinary consultation and postpartum outreach require staff time and reliable referral processes.

Digital health presents both opportunities and implementation challenges. SMS, mobile applications, telehealth, and remote monitoring may reduce travel and facilitate communication and self-management, but implementation depends on digital literacy, technology and connectivity access, and staff capacity. Remote monitoring may reduce some in-person visits while increasing the volume of patient-generated data requiring review. Digital strategies therefore require defined workflows for clinical review, feedback, and treatment escalation, as well as consideration of privacy and data security.

Importantly, resource-constrained settings are heterogeneous. Community health centers, safety-net systems, and rural clinics in high-income countries may face different constraints than low- and middle-income country settings. Given the limited evidence directly evaluating GDM engagement interventions in low-resource settings, the proposed pathway should not be viewed as a fixed package. Rather, its components should be adapted to local workforce, infrastructure, financing, and referral capacity, with more resource-intensive strategies added as feasible. Thus, the pathway is best understood as an adaptable, evidence-informed framework rather than a uniform implementation model.

### 4.3. Translating Evidence into Practice

Although evidence supporting comprehensive stepped care pathways remains limited, the literature identifies four overarching principles for improving patient engagement across the GDM care continuum. These principles synthesize the findings of this review and provide a practical, adaptable framework for resource-constrained settings. The proposed framework is intended to complement, rather than replace, established GDM clinical guidelines, which guide screening, glycemic management, and postpartum testing [1,14]. Its specific contribution is to emphasize how women are linked to care, supported in self-management, engaged through appropriate clinical and digital strategies, and retained through the postpartum transition. Table 3 translates these principles into evidence-informed patient engagement strategies for routine clinical practice; several clinical principles also draw on established GDM clinical guidance and the broader implementation literature where the included studies provided limited direct evidence.

Principle 1. Engage Women Early

Identification of GDM should trigger prompt patient engagement and linkage to care. Women diagnosed with GDM should receive timely follow-up, structured education, and a clear management plan to initiate self-management and minimize delays in treatment. The specific workflow should be adapted to local clinical capacity and resources.

Principle 2. Individualize Care Through Whole-Person Assessment

Effective GDM management requires more than glycemic assessment alone. Early evaluation of health literacy, language needs, social determinants of health, cultural factors, family support, and behavioral health concerns can identify barriers to successful self-management and inform individualized care plans. Whenever possible, assessment should be paired with referrals to appropriate community resources and support services, such as patient navigators, community health workers, food assistance programs, transportation services, behavioral health providers, and culturally appropriate nutrition resources. Identifying barriers alone is unlikely to improve patient engagement unless healthcare teams also help women access the services needed to overcome them.

Principle 3. Support Daily Self-Management

Daily self-management is the foundation of successful GDM care. Structured education, self-monitoring of blood glucose, digital tools, and timely clinician feedback can reinforce healthy behaviors and improve adherence. Digital technologies should complement—not replace—the patient–provider relationship.

Principle 4. Plan Beyond Delivery

Patient engagement should continue beyond pregnancy. Discussing postpartum diabetes screening, arranging the transition to primary care, and using reminder systems before delivery can improve follow-up and support long-term diabetes prevention.

### 4.4. Hypothetical Illustrative Case: Application of the Proposed Pathway

The following hypothetical case is provided solely to illustrate how the proposed principles might be operationalized; it does not represent an evaluated intervention or clinical outcome. A community health center serving a rural district in India provides prenatal care for approximately 1500 women annually, with limited access to endocrinologists and diabetes educators. A 29-year-old woman is diagnosed with GDM through routine antenatal screening at 26 weeks’ gestation. The clinic promptly contacts her and arranges an education visit with a nurse. During the visit, a brief whole-person assessment identifies limited health literacy, financial constraints, and difficulty traveling to the clinic. The patient receives culturally appropriate nutrition counseling, training in self-monitoring of blood glucose, and enrollment in an SMS reminder program. An Accredited Social Health Activist (ASHA) reinforces education during home visits, while the nurse reviews glucose readings weekly and consults the physician when treatment intensification is needed.

Before delivery, the clinic schedules a postpartum oral glucose tolerance test and documents a transition plan for ongoing follow-up through the local primary care clinic. Automated reminders and ASHA outreach encourage attendance at the postpartum visit and reinforce healthy lifestyle behaviors. This hypothetical example illustrates how standardized workflows, multidisciplinary teamwork, and accessible digital communication could be adapted to strengthen patient engagement across the GDM care continuum in a resource-constrained setting. Implementation would nevertheless require appropriate staffing, training, technology, referral capacity, and local infrastructure.

### 4.5. Limitations

This review has several limitations. As a structured narrative review, it did not provide the methodological rigor of a systematic review, and reliance on Consensus as the primary literature identification platform may have resulted in incomplete retrieval. Publication and selection bias are also possible, and heterogeneity across populations, settings, interventions, and outcomes precluded quantitative synthesis.

Formal study-level risk-of-bias and certainty-of-evidence assessments were not conducted; therefore, the descriptive evidence classifications reflect the type and consistency of supporting evidence rather than formal quality ratings. Primary study selection and data extraction were conducted by the first author, with the second author independently verifying a subset, introducing potential reviewer bias. Finally, most evidence evaluates individual components of GDM care rather than the proposed integrated pathway, and evidence from resource-constrained settings remains limited. The pathway should therefore be considered an evidence-informed framework requiring contextual adaptation and prospective evaluation.

### 4.6. Future Research Priorities

Future research should evaluate patient engagement strategies as integrated components of stepped clinical care pathways rather than as isolated interventions. Pragmatic implementation studies are needed to determine how early identification, whole-person assessment, structured education, self-management support, digital engagement, and coordinated postpartum follow-up can be implemented in routine practice, particularly in resource-constrained settings. Priority should also be given to culturally tailored interventions addressing health literacy, language, and digital access; strategies integrating patient navigation and community-based resources; and sustainable models that strengthen postpartum transition and long-term diabetes prevention.

## 5. Conclusions

Effective GDM management requires sustained patient engagement from diagnosis through postpartum care. This review supports a patient-centered approach combining prompt linkage to care, whole-person assessment, culturally responsive education, self-management support, digital engagement with clinician feedback, and coordinated postpartum follow-up. Although evidence for an integrated stepped pathway remains limited, these principles provide a practical framework for improving engagement in low-resource community health settings. Implementation should emphasize accessible, culturally responsive approaches that complement patient–provider relationships and can be adapted to local resources. Further research is needed to evaluate the effectiveness, scalability, and sustainability of integrated patient-engagement pathways in underserved populations.

## Figures and Tables

**Table 1:** Summary Characteristics.

Characteristic	n (%)
Study design	
Systematic review/Meta-analysis/Scoping review	13 (35.1)
Randomized controlled trial	7 (18.9)
Cohort/Observational/cross-sectional study	7 (18.9)
Literature/narrative review	6 (16.2)
Mixed-methods study	2 (5.4)
Intervention development study	1 (2.7)
Interview study	1 (2.7)
Primary evidence domain	
Screening and early identification	15 (40.5)
Patient engagement strategies	12 (32.4)
Postpartum transition and diabetes prevention	5 (13.5)
Whole-person assessment	5 (13.5)
Country or setting	
International/Multinational	20 (54.1)
United States	5 (13.5)
United Kingdom	3 (8.1)
Portugal	2 (5.4)
China	2 (5.4)
Ireland	1 (2.7)
Iran	1 (2.7)
Nigeria	1 (2.7)
Thailand	1 (2.7)
Netherlands	1 (2.7)

**Table 2:** Summary of Included Studies and Key Findings.

Study	Study Design	Domain	Key Finding(s)
Ali (2025) [19]	Systematic review	1. Screening & early identification	Early screening for GDM (20 weeks) does not improve maternal or neonatal outcomes and may lead to additional interventions without clear benefit.
Ardabili (2026) [20]	Systematic review	2. Whole-person assessment	Patient-centered, culturally sensitive strategies integrating individual, interpersonal, and systemic elements are crucial for effective GDM management.
Bendsen (2025) [21]	Randomized controlled trial	3. Patient engagement strategies	Pregnant women using the Liva app show diverse engagement patterns, highlighting the need for tailored support and tailored interventions in digital health during pregnancy.
Benham (2023) [22]	Systematic review	1. Screening & early identification	Precision medicine approaches, using routine clinical measures, could enable earlier identification of women needing pharmacological therapy for GDM.
Birati (2022) [23]	Systematic review	3. Patient engagement strategies	Mobile health and telemedicine systems can improve glycemic control and diabetes self-management for pregnant women with GDM, but more research is needed on their cultural and digital health literacy appropriateness.
Bose Brill (2022) [24]	Cohort study	4. Postpartum transition & T2D prevention	Participation in the Mother–Infant Dyad postpartum primary care program was associated with higher rates of T2D screening, postpartum visit attendance, and prediabetes diagnosis.
Davis (2021) [25]	Randomized controlled trial	1. Screening & early identification	The IADPSG screening criteria resulted in more women diagnosed with gestational diabetes than Carpenter–Coustan without reducing the incidence of large-for-gestational-age birth weight or maternal or neonatal morbidity.
Edwards (2022) [16]	Systematic review	3. Patient engagement strategies	Mobile health interventions can improve GDM management and reduce long-term complications, but further development is needed for prevention of recurrence and type 2 diabetes.
Fiskå (2023) [26]	Systematic review	4. Postpartum transition & T2D prevention	There is no established systematic cardiovascular follow-up strategy for women after GDM, and eHealth technologies show no consistent, statistically significant advantage over conventional follow-up.
Griffin (2000) [27]	Randomized controlled trial	1. Screening & early identification	Universal screening for GDM detects more cases, facilitates early diagnosis, and is associated with improved pregnancy outcomes compared to risk factor-based screening.
Guo (2019) [28]	Randomized controlled trial	3. Patient engagement strategies	Mobile health intervention improves compliance, blood glucose control, and reduces weight gain in patients with GDM, reducing complications during pregnancy.
Hillier (2021) [29]	Randomized controlled trial	1. Screening & early identification	The one-step screening approach detects more GDM than the two-step approach, but there are no significant differences in risks of perinatal and maternal complications.
Hosseini (2018) [30]	Cohort study	1. Screening & early identification	The two-step approach detects GDM more effectively and has a stronger impact on adverse pregnancy outcomes than the one-step approach.
Lee (2019) [31]	Cohort study	1. Screening & early identification	The 1-step approach for GDM may reduce the risk of large for gestational age infants, but its cost-effectiveness remains unclear compared to the 2-step approach.
Luo (2025) [32]	Systematic review	4. Postpartum transition & T2D prevention	GDM requires precision-based, life-course care, with lifestyle modification, pharmacologic agents, and postpartum follow-up being crucial for long-term metabolic health.
Matta-Coelho (2019) [33]	Cohort study	1. Screening & early identification	Universal screening for GDM in Portugal reduces obstetric and neonatal complications, but risk-based screening may miss one-third of women with risk factors.
Mclennan (2024) [34]	Cohort study	1. Screening & early identification	During COVID-19, new diagnostic criteria identified more women with GDM but had minimal impact on pregnancy outcomes.
Mobin (2025) [35]	Literature review	2. Whole-person assessment	Women with GDM prefer personalized, flexible management plans that consider lifestyle, healthcare professionals, and family involvement, to improve adherence and health outcomes.
Mohamed (2025) [36]	Scoping review	3. Patient engagement strategies	Digital health technologies positively impact antenatal care by managing clinical conditions, enhancing knowledge, promoting birth preparedness, and reducing financial burdens for patients and healthcare systems.
Moon (2022) [37]	Literature review	1. Screening & early identification	The one-step approach for diagnosing GDM has increased the incidence of the condition, but its clinical benefit in reducing adverse pregnancy outcomes remains controversial.
Morampudi (2017) [38]	Literature review	3. Patient engagement strategies	In India, managing GDM is challenging due to conflicting guidelines and treatment protocols, and a collaborative approach with patient compliance and education is crucial for better pregnancy outcomes.
Murphy (2023) [39]	Intervention development study	2. Whole-person assessment	The IINDIAGO intervention was developed using the Behaviour Change Wheel and COM-B model to address informational and psychosocial needs through peer counseling, diabetes nursing, and integrated postpartum follow-up.
Nielsen (2014) [40]	Systematic review	2. Whole-person assessment	GDM screening, treatment, and follow-up remain under-prioritized, with self-efficacy and social support being key determinants in improving compliance.
Nunes (2025) [41]	Mixed methods study	3. Patient engagement strategies	The NUTRIA Toolkit, a user-centered, evidence-based digital toolkit, effectively supports self-care in GDM by providing behavioral and educational content, particularly on nutrition.
Nwali (2021) [42]	Cross-sectional study	1. Screening & early identification	Universal screening identified more GDM cases than selective risk-factor screening, which missed 31.11% of cases.
Ohene-Agyei (2024) [14]	Systematic review	4. Postpartum transition & T2D prevention	Most postnatal care recommendations for GDM are not based on high certainty evidence, and further research is needed to improve long-term maternal health.
Payne (2026) [43]	Interview study	2. Whole-person assessment	Women with GDM often have limited knowledge of the condition before pregnancy, and need clear communication during pregnancy and targeted interconception support.
Phoblap (2024) [44]	Randomized controlled trial	1. Screening & early identification	The one-step method of universal screening for gestational diabetes mellitus significantly increases the prevalence of GDM by nearly three times that of the two-step method, potentially increasing care costs and burdens without clear benefits.
Qaderi (2024) [45]	Systematic review	1. Screening & early identification	During the COVID-19 pandemic, gestational diabetes screening and management strategies shifted from OGTTs to HbA1c assays, with medical nutrition therapy being the primary management strategy.
Raets (2021) [46]	Narrative review	1. Screening & early identification	Early pregnancy screening for GDM is important, but the benefits and effectiveness of screening and treating GDM in early pregnancy remain unclear.
Roberts (2021) [47]	Mixed methods study	4. Postpartum transition & T2D prevention	Women with gestational diabetes mellitus in pregnancy in the UK experience dissatisfaction with postnatal care due to inconsistencies in information and advice.
Safiee (2022) [48]	Systematic review	3. Patient engagement strategies	eHealth technologies can improve GDM self-management, but challenges include usability and privacy concerns, while convenience, motivation, and consistent monitoring are facilitators.
Tian (2021) [13]	Randomized controlled trial	3. Patient engagement strategies	Telemedicine, such as WeChat group chats, is more effective for blood glucose control than standard clinical prenatal care in women with gestational diabetes mellitus.
Tieu (2017) [49]	Systematic review	1. Screening & early identification	Universal screening for GDM leads to more diagnoses, while low-quality evidence suggests no clear differences between primary care and secondary care screening for maternal and infant outcomes.
van den Berg (2025) [50]	Cohort study	3. Patient engagement strategies	Redesigning gestational diabetes mellitus care pathways can improve value and patient-reported experiences while maintaining clinical outcomes and achieving cost savings.
Xega (2025) [51]	Narrative review	3. Patient engagement strategies	Chrononutrition, adjusting meal timing based on real-time glucose patterns, could improve maternal glycemic control and reduce the need for pharmacotherapy, potentially benefiting both mother and child.
Zork (2022) [52]	Narrative review	3. Patient engagement strategies	Telehealth can improve convenience, efficiency, and communication in diabetes management for pregnant women, potentially leading to better outcomes.

**Table 3:** Patient Engagement Strategies Across the GDM Care Pathway.

Pathway Step	Patient Engagement Strategy	Supporting Evidence	Key Supporting Evidence	Practical Implementation in Low-Resource Settings
Early Identification	Rapid linkage to care following diagnosis	Primarily observational, qualitative, or implementation evidence	Universal screening detects more GDM but improved outcomes depend on timely management	Contact patients promptly following diagnosis and schedule education and follow-up
Whole-Person Assessment	Assess SDOH, literacy, language, mental health, family support	Primarily observational, qualitative, or implementation evidence	Qualitative studies consistently identify these as major barriers	Use a brief standardized intake tool completed by nurse or CHW
Structured Education	Deliver culturally appropriate diabetes education immediately after diagnosis	Multiple systematic reviews and/or RCTs	Multiple RCTs and reviews demonstrate improved knowledge, adherence, and glycemic control	Standardized education visit with teach-back and written materials
Lifestyle Coaching	Collaborative goal-setting for nutrition and physical activity	Multiple systematic reviews and/or RCTs	Lifestyle intervention is first-line therapy and effective for most women	Individualized goals reinforced at each visit
Self-Monitoring	Teach SMBG with rapid review of readings	Multiple systematic reviews and/or RCTs	Strong evidence supports SMBG as part of comprehensive management	Review logs weekly using nurse or diabetes educator
Digital Engagement	SMS, apps, telehealth, digital coaching	Systematic reviews and multiple primary studies	Consistent improvements in adherence, engagement, and satisfaction	Use lowest-cost technology patients can reliably access
Active Clinical Feedback	Review transmitted glucose values and adjust therapy promptly	Primarily observational, qualitative, or implementation evidence	Interactive monitoring outperforms passive data collection	Establish standing protocols for clinician review
Medication Intensification	Shared decision-making regarding insulin or metformin	Multiple systematic reviews and/or RCTs	Strong evidence supports timely escalation when lifestyle therapy fails	Use decision aids addressing patient preferences and concerns
Multidisciplinary Coordination	Integrate obstetrics, nursing, dietetics, behavioral health, CHWs	Systematic reviews and multiple primary studies	Better care coordination and patient experience	Regular interdisciplinary case review for complex patients
Postpartum Transition	Schedule testing before delivery and provide reminder outreach	Primarily observational, qualitative, or implementation evidence	Strong evidence identifies postpartum care as the greatest gap	Arrange postpartum OGTT appointment before hospital discharge

## Data Availability

No new data were created or analyzed in this study. Data sharing is not applicable to this article.

## Supplementary Materials

The following supporting information can be downloaded at: https://www.mdpi.com/article/10.3390/healthcare14172860/s1.
