# Supplementary material for: Patient Engagement Strategies for Women with Gestational Diabetes Mellitus in Low-Resource Community Health Settings: A Narrative Review and Clinical Principles
Source: Healthcare (Basel). 2026 Sep 5;14(17):2860. doi: 10.3390/healthcare14172860 (PMC13566134; doi:10.3390/healthcare14172860)
Supplement: Supplementary file 1 [file healthcare-14-02860-s001.zip › healthcare-4506515-supplementary.pdf]

Search Stage	Natural-Language Search Query / Search Action
Original Deep Search prompt	What is the evidence for a stepped clinical care pathway for women with gestational diabetes mellitus (GDM)? Review four domains separately: (1) universal vs. risk-based screening, focusing on detection rates and maternal/neonatal outcomes; (2) whole-person assessment of SDOH, cultural, language, psychosocial, and access barriers that affect treatment adherence; (3) digital engagement strategies, distinguishing interactive telehealth, remote monitoring with clinician feedback, reminders, and educational tools from passive monitoring alone; and (4) postpartum transition interventions that improve postpartum glucose testing, primary care linkage, and prevention of type 2 diabetes. Prioritize systematic reviews, randomized trials, implementation studies, and qualitative studies; summarize the strength of evidence, where findings are mixed, and practical implications for low-resource care settings.
Citation expansion	Citation graph search using 50 seed publications.
Domain-specific search	Universal versus risk-based screening for gestational diabetes detection and maternal neonatal outcomes
Domain-specific search	Social determinants, cultural, language, psychosocial, and access barriers in gestational diabetes care
Domain-specific search	Digital engagement strategies for gestational diabetes: telehealth, remote monitoring, education, reminders
Domain-specific search	Postpartum interventions for gestational diabetes: glucose testing, primary care linkage, type 2 diabetes prevention
Guidelines/frameworks	Clinical guidelines for stepped care pathways in gestational diabetes mellitus
Guidelines/frameworks	Consensus statements and models for gestational diabetes screening and management
Guidelines/frameworks	Foundational frameworks for gestational diabetes care pathways
Alternative terminology	Tiered management approaches for gestational diabetes mellitus
Alternative terminology	Social determinants and cultural barriers in GDM care adherence
Alternative terminology	Mobile health interventions and telemedicine for gestational diabetes postpartum follow-up
Alternative terminology	Graduated care models and transition interventions after gestational diabetes
Limitations/contradictory evidence	Limitations of stepped care pathways in gestational diabetes management
Limitations/contradictory evidence	Critiques and barriers to digital health interventions for gestational diabetes
Limitations/contradictory evidence	Null findings and challenges in postpartum interventions for women with gestational diabetes
Limitations/contradictory evidence	Mixed evidence on universal versus risk-based screening for gestational diabetes outcomes
Related care models	Stepped care pathways in type 2 diabetes management
Related care models	Digital health interventions for hypertension stepped care
Related care models	Chronic disease management stepped care models and digital engagement

Low-resource implementation	Implementation of stepped gestational diabetes care pathways in low-resource settings
Low-resource implementation	Barriers and facilitators to gestational diabetes management in low-income or resource-limited contexts
Low-resource implementation	Adaptations of digital health interventions for gestational diabetes in low-resource environments
